# IntelliPatent: a web-based intelligent system for fast chemical patent claim drafting

**DOI:** 10.1186/s13321-019-0401-4

**Published:** 2019-12-11

**Authors:** Pei-Hua Wang, Yufeng Jane Tseng

**Affiliations:** 1 Graduate Institute of Biomedical Electronics and Bioinformatics, National Taiwan University, No. 1 Sec. 4, Roosevelt Road, Taipei, 106 Taiwan; 2 Department of Computer Science and Information Engineering, National Taiwan University, No. 1 Sec. 4, Roosevelt Road, Taipei, 106 Taiwan

**Keywords:** Web server, Markush structure, Pharmaceutical patent

## Abstract

The first step of automating composition patent drafting is to draft the claims around a Markush structure with substituents. Currently, this process depends heavily on experienced attorneys or patent agents, and few tools are available. *IntelliPatent* was created to accelerate this process. Users can simply upload a series of analogs of interest, and *IntelliPatent* will automatically extract the general structural scaffold and generate the patent claim text. The program can also extend the patent claim by adding commonly seen R groups from historical lists of the top 30 selling drugs in the US for all R substituents. The program takes MDL SD file formats as inputs, and the invariable core structure and variable substructures will be identified as the initial scaffold and R groups in the output Markush structure. The results can be downloaded in MS Word format (.docx). The suggested claims can be quickly generated with *IntelliPatent*. This web-based tool is freely accessible at https://intellipatent.cmdm.tw/.

## Introduction

Claims are the most important sections in composition patents [1]. In the pharmaceutical industry, claims should include key compounds and all structural derivatives that are likely to have the same effects [2]. This is achieved by Markush structures [3] that describe a series of chemical compounds having a common core structure and variable substituents called R groups [4].

To ensure the novelty of a new composition patent, one needs to make sure that no compound in the claims is taken by prior arts. One common routine is to perform structure searches in chemical databases [5]. One of the most useful free online databases for chemical prior-art search is SureChEMBL [6]. Performing a structure search using the Markush structure as input is the most efficient method. To generate the input Markush structure, one needs to study the compounds they want to patent to identify the input scaffold and R group variations.

Previous studies focused on interpretation or searching of Markush structure. For an example, MarVis is capable of visualizing and analyzing Markush structures from a composition patent [7]. Its web based application, iMarVis, revised the underlying R group numbering system to deal with nested R group presentation [8]. An algorithm based on SMIRK language is introduced to solve the query for a compound within a Markush structure [9].

The next step in drafting compositional patent claims is to maximize patent coverage. The algorithm implemented in ChemAxon can automatically generate Markush structures [10]. Periscope system helps generating Markush structures from compounds, visualization, and searching specific chemical structures [11]. However, both systems cannot expand the coverage by adding new variations to the Markush structure. If the patent coverage is fully maximized, a monopoly state of first-in-class drugs can be achieved and profits will be ensured [12, 13].

Adding variations beyond the current Markush structure relies on the experience of patent attorneys. Hence, patent coverage becomes more or less dependent on the writers. Despite the advance in chemical informatics, drafting and describing Markush structures still requires substantial manual effort. In this study, we introduce a publicly available server, *IntelliPatent*, for rapid chemical patent drafting that includes the option to recommend Markush structures to expand composition patent claims. With an R group library built from 30 composition patents, the definition of output Markush structure can be extended based on the input compounds.

## Implementation

### Dataset

A library containing 269 R groups was constructed from 30 patents of the top-selling drugs in the US in 2005. The compounds in the “Biological Study” or “Preparation” sections were retrieved from SciFinder with the patent IDs listed in the study by Hattori et al. [14]. The structures of the 30 drug entities were downloaded from DrugBank [15] using the trade names.

### Building the R group library

The scaffolds of the 30 drug entities were identified using Scaffold Hunter-2.4.1 [16]. Using JChem Base API [17], the scaffolds of the compounds were removed to obtain 5589 structural fragments, which were saved as R groups. We removed 5183 duplicates including optical isomers and filtered out 2 R groups having deuterium, 2 having phosphorus and 133 having aliphatic carbon chains with lengths longer than 6 atoms to have the final 269 R groups.

Next, we identified 28 common groups along with their text terms from the patent claims. The common groups include functional groups with defined structures (e.g., hydroxyl, cyano and halo) and general groups that incorporate a series of structures (e.g., alkyl, aryl, and heteroaryl). The SMARTS notations for detecting the common groups were generated to categorize the R groups in the library into 57 categories, including composite categories such as alkyl–aryl. The common groups, which are defined as the substituents on carbon chains or rings, were also recorded.

### R groups from approved drugs

The structures of 2413 approved drugs were retrieved from DrugBank on 2019/11/19. For those structures with more than one molecules, only the compound with the highest molecular weights were retained. We filtered out big molecules (MW > 800 Da), tiny molecules (MW < 100 Da and molecules with less than 6 carbon atoms) and duplicates including optical isomers to have 1921 drugs. The R groups from 1808 drugs were obtained by removing the scaffolds from compounds as the methods we used in building R group library in this work. There are 364 different R groups from approved drugs.

### Expanding the coverage of the Markush structure

The scaffold of the initial Markush structure, including the scaffold and collections of R groups attached to the same atoms, is generated by RDKit using the *FindMCS* function from the input compounds. For every R group structure, the presence of common R groups will be detected. If the R group comprises several common groups, the text term describing the R group will be generated from the most distant common group moving to the ones near the attachment point. For R groups attached to a carbon chain or ring in the scaffold, if the R group structure exactly matches any of the common substituents on the corresponding main structure in our library, the rest of the common substituents will be added to the same ring or carbon chain.

### Case study data

To demonstrate how *IntelliPatent* works, the chemical composition patents of bromazine, orphenadrine, omeprazole and timoprazole were retrieved from the European Patent Office using patent IDs US2527963A, US2567351A, EP0005129B1 and US4045563A, respectively. The example compounds for bromazine and omeprazole and the Markush structures of the subsets from the claims of orphenadrine and timoprazole were constructed manually.

### User’s privacy

The input file from user is deleted immediately after computation at the end of the session. Linux *crontab* command was used to check all the output files twice a day and remove the files that were created more than 12 h ago. Therefore, the output data including graph, text and MS Word files will be deleted in less than a day. Since the server does not generate or retain any log file, the IP address of users will not be traceable via the server of *IntelliPatent*. All web traffic is encrypted via HTTPS.

## Results and discussion

### R group library analysis

269 R groups are generated using the example compounds from the patents of 30 top-selling drugs and their scaffolds were processed by Scaffold Hunter, based on the concept that compounds in a Markush structure can be divided into a scaffold plus several R groups.

The top 10 R groups and their percentage of counts in all the 218 R groups showing up at least twice among all 269 groups are shown in Table 1. The top duplicate R group, methyl, alone accounts for nearly 20% of occurrence. The top 10 groups account for 63% among all the duplicate R groups and they are all small groups containing less than 5 atoms. These 10 top duplicate R groups can be considered basic sets in R group variations when drafting patent claims.

**Table 1:** The top 10 R groups in library

R group	SMILES	Occurrence (%)
Methyl	*C	19.9
Fluoro	*F	8.4
Methoxy	*OC	7.0
Hydroxy	*O	6.3
Sulfamoyl	*S(=O)(=O)N	5.6
Chloro	*Cl	5.1
Hydrogen	*H	3.8
Ethynyl	*C#C	2.8
Trifluoromethyl	*C(F)(F)F	2.2
Carboxy	*C(=O)O	1.9

To see how much those R groups from top 30 best selling drugs overlapped with the DrugBank records, we extracted and analyzed the R groups of 1808 approved drugs from DrugBank. There are 364 different R groups from approved drugs. After comparison with our R group library, we found that 84.8% of the R groups from approved drugs are covered by our library. The top 10 R groups with the highest occurrence from approved drugs is shown in Table 2.

**Table 2:** The top 10 most used R groups from approved drugs

R group	SMILES	Occurrence (%)	Both top 10
Methyl	*C	23.6	O
Hydroxy	*O	18.2	O
Chloro	*Cl	6.4	O
Amino	*N	5.1	
Methoxy	*OC	5.0	O
Carboxy	*C(=O)O	3.9	O
Fluoro	*F	2.9	O
Ethyl	*CC	2.2	
Hydroxymethyl	*CO	1.7	
Trifulromethyl	*C(F)(F)F	1.0	O

There are 7 groups which appear in both of the top 10 R lists. This demonstrates that our R group library cover most of the R groups from approved small molecular drugs.

### Case study 1: bromazine

**Fig. 1:**
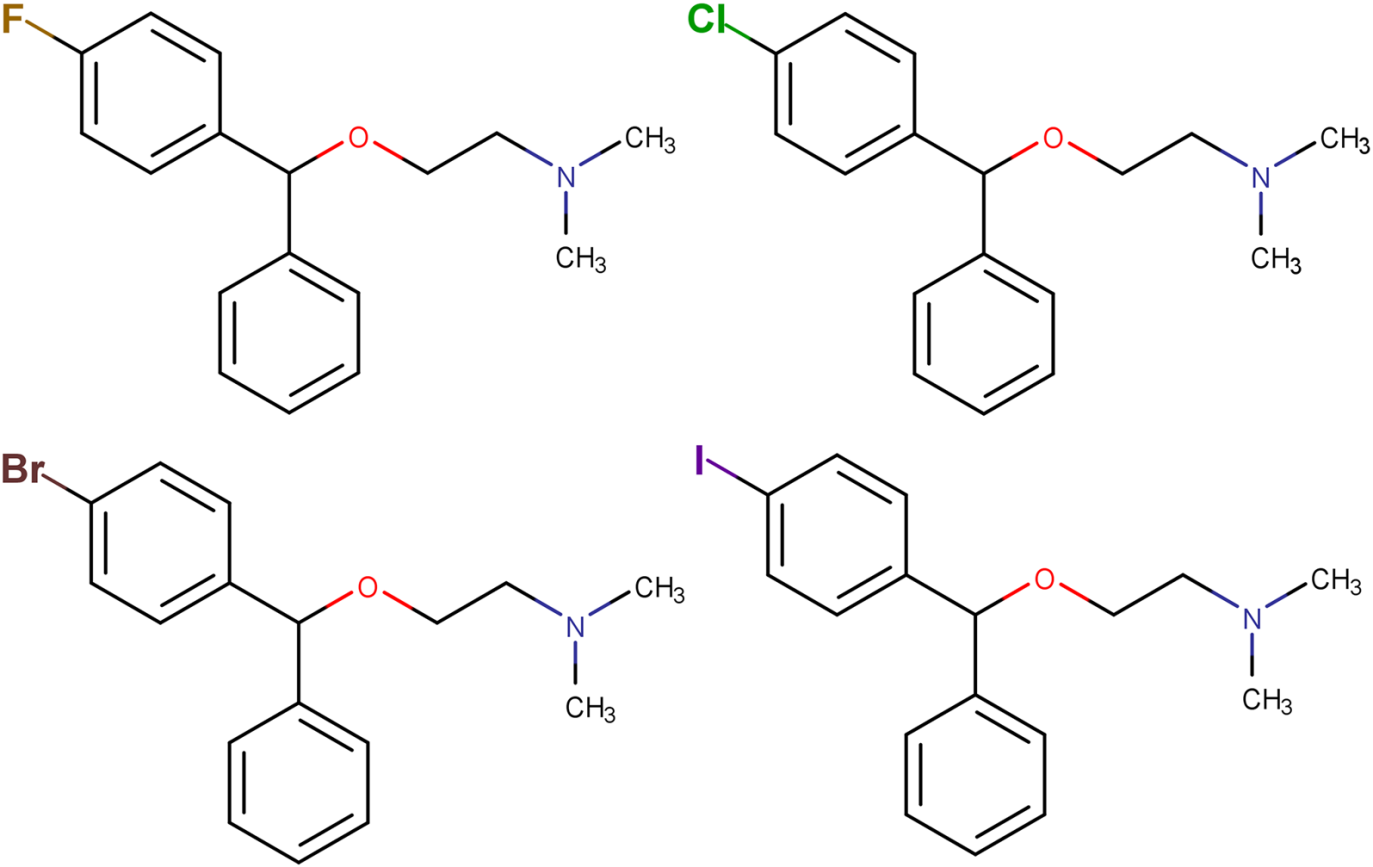
The chemical structures of compounds generated from the composition patent of bromazine (US2527963A) for demonstration

**Fig. 2:**
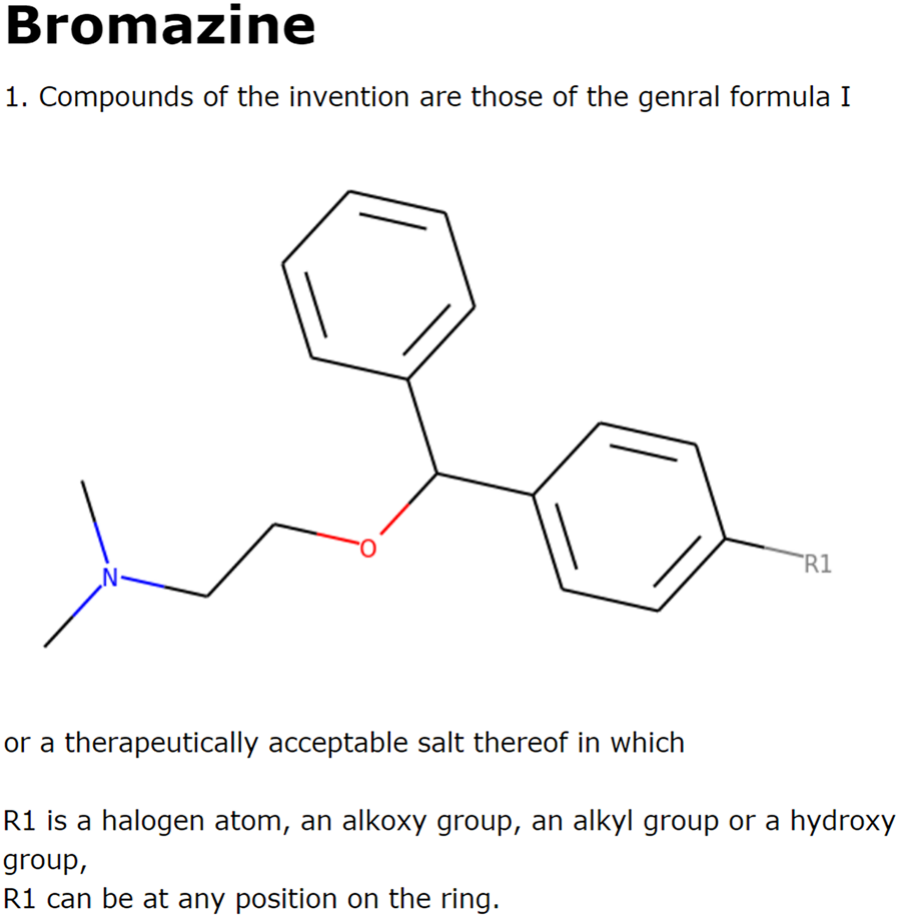
The output claims using the four example compounds from the patent of bromazine (US2527963A)

We used bromazine, a first-generation antihistamine, to demonstrate how *IntelliPatent* generates patent claims for a series of input compounds. Bromazine was patented in 1950, and the composition patent contained only four compounds (see Fig. 1). The resulting claims contained a scaffold automatically generated from all the input compounds and several additional R groups and position variations with the corresponding text (Fig. 2).

**Fig. 3:**
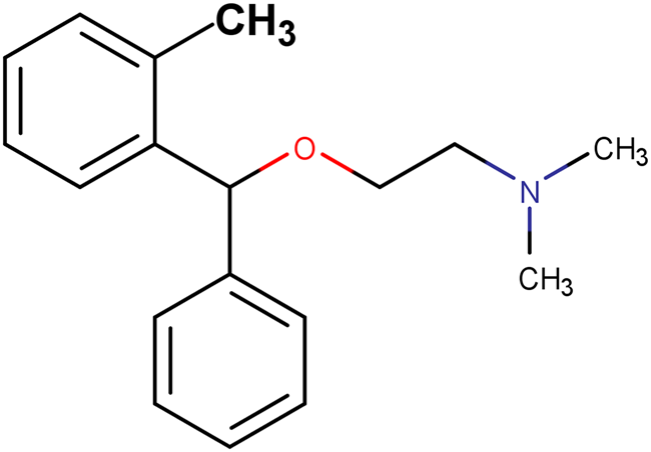
The active moieties of orphenadrine

**Fig. 4:**
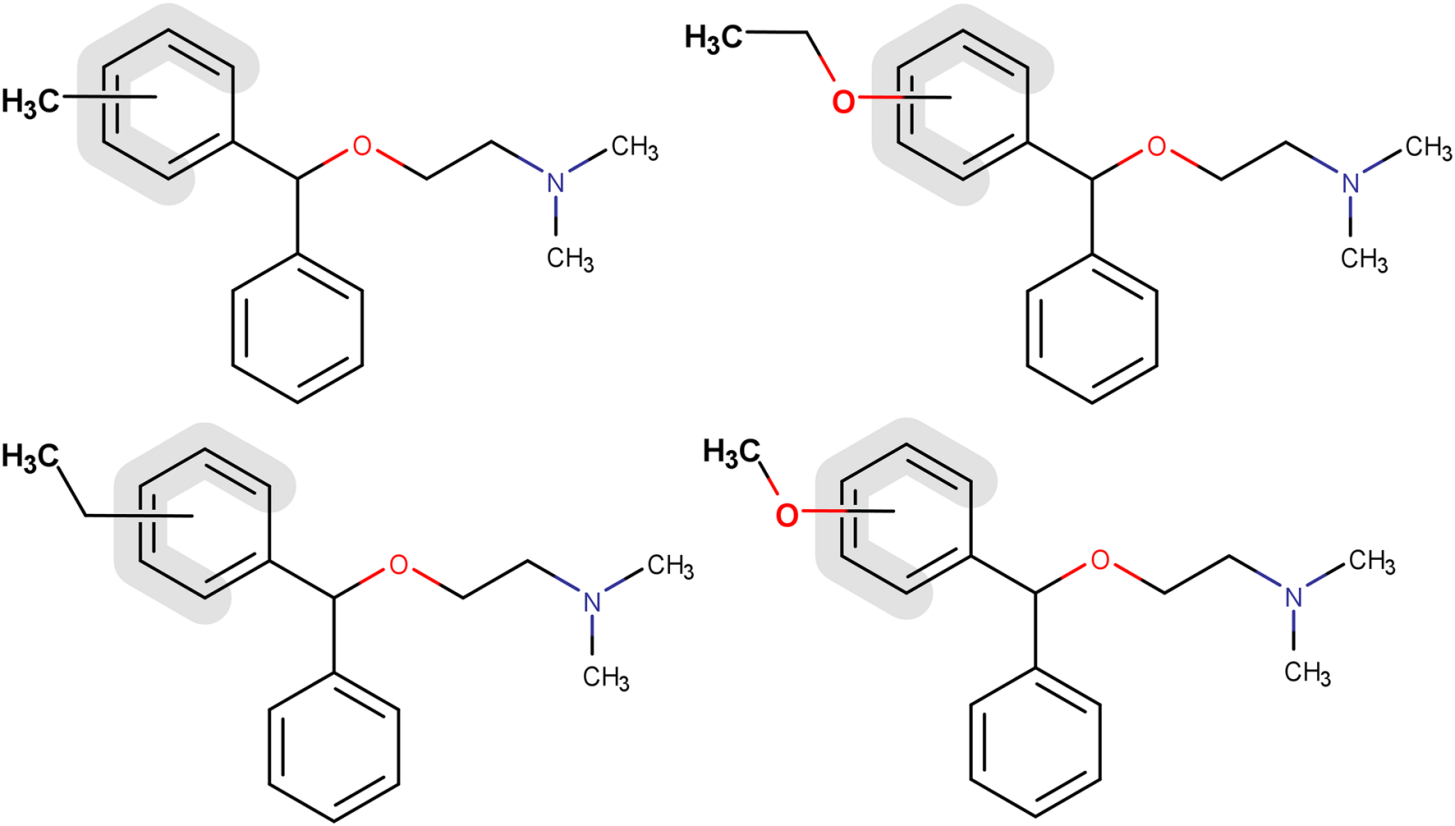
The subset of structures in the claims of orphenadrine that were covered by output claims. The input structures were the example compounds in the patent of bromazine

This output shows another benefit and potential use for *IntelliPatent*, namely, broader coverage of the patent. The output claims from *IntelliPatent* using the bromazine compounds successfully covered the active moieties of orphenadrine (Fig. 3), an antihistamine of the same class patented in 1951, one year after the patent of bromazine. The four sets of compounds claimed in the patent of orphenadrine that are also covered by the output claims are shown in Fig. 4. Hence, *IntelliPatent* is capable of generating Markush structures for a series of analogs and expanding patent coverage by adding appropriate variations. The output claims can serve as a checklist to assist patent attorneys in designing claims.

### Case study 2: omeprazole

**Fig. 5:**
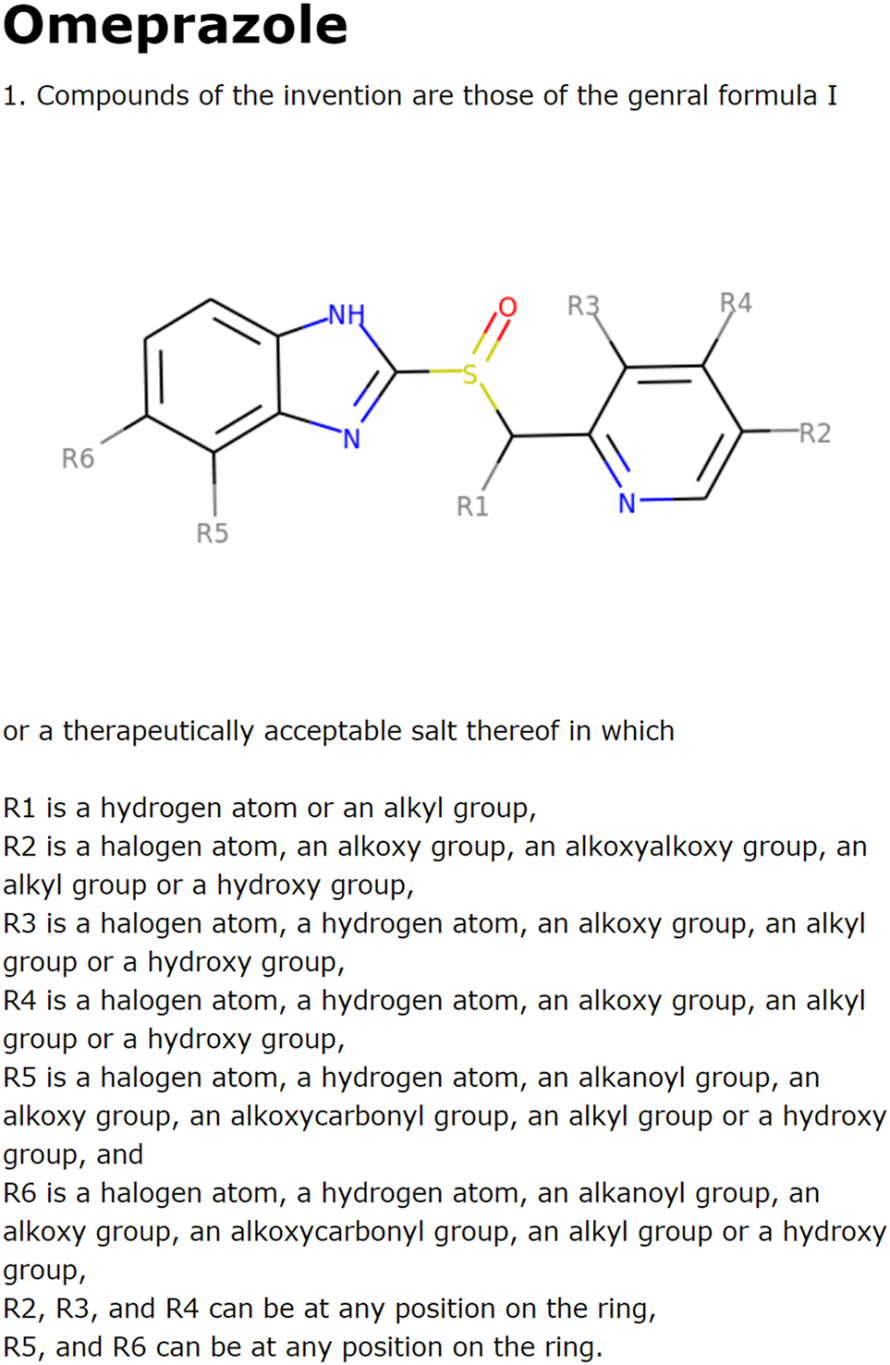
The output claims using the 30 example compounds from the patent of omeprazole (EP0005129B1)

The second drug for demonstration is omeprazole, the first clinically used proton pump inhibitor [18]. Omeprazole was patented in 1979, and there are 30 example compounds in the patent (see Additional file 1 for the structures). We use the 30 example compounds as input for *IntelliPatent* and the generated patent claims (see Fig. 5) covered all the compounds claimed in the patent of omeprazole.

**Fig. 6:**
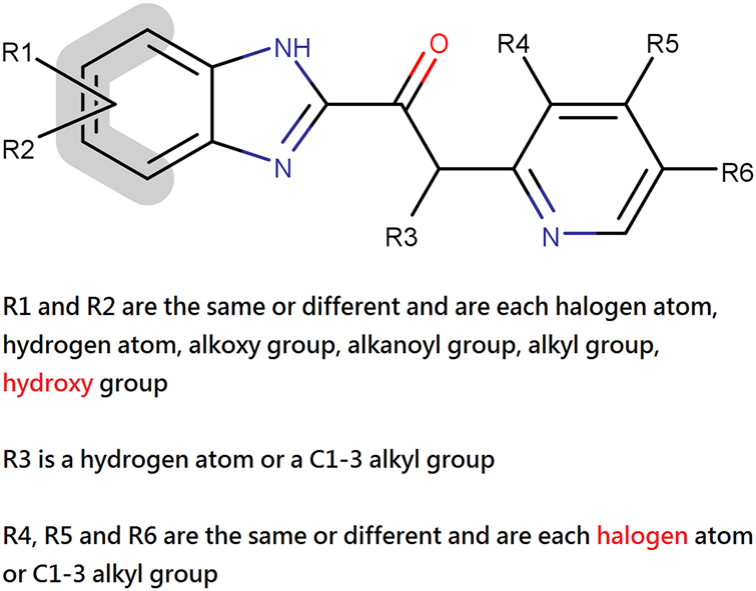
The claims from timoprazole that were covered by the output using example compounds from omeprazole. The original patent claims of omeprazole (EP0005129B1) did not include the structures shown in the figure. The input structures to IntelliPatent are the example compounds in the patent of omeprazole. The two critical R groups that were absent in the original patent but present in our resulting claims are highlighted in red

Our output claims include part of the claims from timoprazole, a proton pump inhibitor of the same class patented in 1977. The compounds from the patent of timoprazole that were covered by our generated claims, but not by those from omeprazole, are shown in Fig. 6. The two critical R groups that were absent in the original patent but present in our resulting claims are the hydroxy group on the benzimidazole and halogen substituents on pyridine; the two critical R groups are highlighted in red in Fig. 6. If *IntelliPatent* were used in patent drafting of omeprazole, the patent coverage would be properly extended and these compounds could not be claimed by future patents. This implies that *IntelliPatent* is also useful after the introduction of Markush structure in pharmaceutical composition patents.

### Web server

**Fig. 7:**
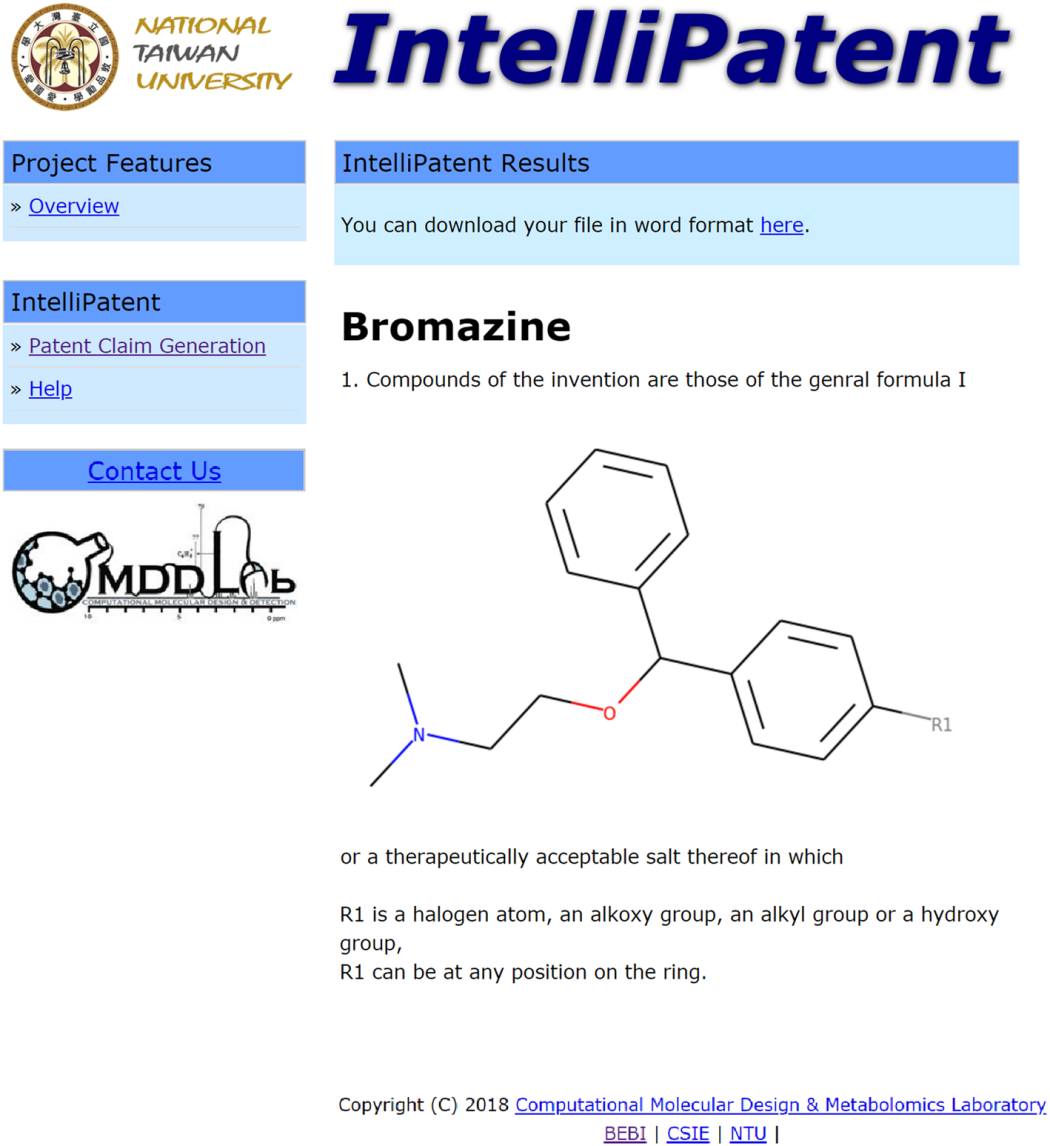
The result page of *IntelliPatent* using the four example compounds from the patent of bromazine

*IntelliPatent* is simple and intuitive to use, and users only need to upload and submit their compounds to use this service. Input file is accepted as MDL SD file, and the file should contain more than one compound that shares the same core structure. The output will be the patent claims with Markush structures and the R group definition text. The R group variations will be broadened with the built-in R group library. Figure 7 shows the results page generated using the compounds in the composition patent of bromazine in Fig. 1 as input. The chemical scheme of the Markush structure is shown after the first sentence of the claims, and the definition of the R groups follows. The results can be downloaded in MS format by clicking the hypertext at the top of the result page.

## Conclusions

*IntelliPatent* is a freely accessible web server that can automatically generate patent claims from input compounds and further expand claim coverage using an R group library extracted from 30 patents. The resulting claim can serve as a first draft to reduce the human labor required to determine a suitable Markush structure from dozens or even hundreds of compounds. The results can also be used as a checklist while drafting claims to avoid omitting common variations in R groups that should be covered and protected. We hope that *IntelliPatent* can mitigate the heavy workload of patent attorneys and give scientists some insights into their compounds and the corresponding claims.

## Availability and requirements

Project name: IntelliPatent.

Project home page: https://intellipatent.cmdm.tw/.

Operating system(s): Platform independent.

Programming language: Python.

Other requirements: None.

License: None.

Any restrictions to use by non-academics: None.

## Supplementary information

**Additional file 1.** Example compounds in the composition patent of omeprazole. The file contains all the structures of 30 example compounds from the patent of omeprazole (EP0005129B1) in MOL SD file format. This file was used as input in the case study of omeprazole.

## Availability of data and materials

The tool is freely available at https://intellipatent.cmdm.tw/.
